# Primary Grocery Shopping Location is Associated With Vegetable Consumption in Underserved Communities

**DOI:** 10.1016/j.jneb.2026.02.013

**Published:** 2026-04-24

**Authors:** Lucia A. Leone, Anne Lally Mathiebe, Christina Kasprzak, Lindsey Haynes-Maslow, Samina Raja, Leah N. Vermont, Laurene Tumiel-Berhalter, Rocco Paluch, Alice Ammerman

**Affiliations:** 1 Department of Community Health and Health Behavior, School of Public Health and Health Professions, University at Buffalo, Buffalo, NY; 2 Department of Health Policy and Management, Department of Nutrition, Gillings School of Global Public Health, University of North Carolina at Chapel Hill, Chapel Hill, NC; 3 Department of Urban and Regional Planning, School of Architecture and Planning, University at Buffalo, Buffalo, NY; 4 Department of Family Medicine, Jacobs School of Medicine and Biomedical Sciences, Buffalo, NY; 5 Division of Behavioral Medicine, Department of Pediatrics, Jacobs School of Medicine and Biomedical Sciences, University at Buffalo, Buffalo, NY; 6 Department of Nutrition, Gillings School of Global Public Health, Center for Health Promotion and Disease Prevention, University of North Carolina at Chapel Hill, Chapel Hill, NC

**Keywords:** food shopping, fruit and vegetable intake, food security, food retail

## Abstract

**Objective:**

This paper characterizes the relationship between primary grocery shopping location (i.e., primary store type) and food security status, fruit and vegetable consumption, and perceptions of the food environment.

**Methods:**

Survey data from shoppers in predominantly lower-income urban areas were analyzed to identify associations with primary store type (supermarket, supercenter, buying club, or small grocery store).

**Results:**

Among respondents (n = 698), most reported a supermarket as their primary store type (67.5%). There were no associations between primary store type and food security or fruit consumption, but vegetable intake was higher for small grocery store (2.00 times/d) and supermarket customers (1.82 times/d) compared with supercenter shoppers (1.52 times/d, *P* = 0.006 and 0.04, respectively). Small stores were also rated as having more affordable produce than supermarkets (*P* < 0.001), and perceived affordability was associated with higher vegetable intake (*P* = 0.009).

**Conclusions and Implications:**

Understanding shoppers’ perceptions of food stores may help identify targets for improving diet.

## INTRODUCTION

In 2022, 17 million individuals in the US (12.8%) lived in food-insecure households.^1^ Food insecurity rates were substantially higher for Black (22.4%) and Hispanic (20.8%) households and those with incomes below 185% of the federal poverty level (32.0%). Food insecurity has been associated with lower consumption of healthy foods, such as fruits and vegetables (F&Vs).^2,3^ In 2019, only 6.8% of individuals with lower income reported meeting F&V recommendations.^4^ Fruits and vegetables consumption is an important component of disease prevention. Adults who consume more F&V are less likely to develop heart disease, type 2 diabetes, certain types of cancer, and are more likely to sustain a healthy weight.^5–7^ In the US, substantial socioeconomic disparities in chronic disease prevalence are driven by poor diets among Americans with lower incomes.^8,9^ These dietary disparities are thought to be caused by limited access to fresh F&V, coupled with a greater prevalence of fast-food outlets in minority communities with lower income.^10,11^ Perceived lower consumer demand for healthy foods in combination with high costs of operations in certain markets impacts the economic decisions of retailers and can contribute to differential access through real or perceived market failure.^12,13^ However, market factors cannot solely account for access disparities, as neighborhoods impacted by historical racism and redlining practices are more likely to experience lower healthy food access.^14,15^

Several types of food retail interventions have emerged to help address disparities in food access with the goal of improving diet and/or food insecurity.^16^ Interventions have mainly focused on (1) opening supermarkets in underserved areas,^17^; (2) improving healthy food availability in corner and convenience stores,^18^ and (3) creating alternative food sources like mobile produce markets or home delivery.^19,20^ However, these approaches have faced several challenges. First, research has shown that supermarket and corner store interventions have impacted food availability, but they have mixed or no affect on dietary quality and have not produced any significant improvements in F&V intake.^16,18,21^ Second, interventions to improve the availability, quality, and variety of produce in corner or convenience stores often suffer from logistical challenges (supply chain issues; minimum purchasing requirements, lack of space/cool storage, etc.) and mistrust between community members and store owners.^18,22^ Third, mobile produce markets, or mobile markets, have shown promise for improving F&V consumption^23^ and reducing food insecurity^24^ and are well received by community members,^22,25^ but their reach can be limited.^19^ In addition, online shopping with home delivery is a newer option for communities with lowe income, but research on its impact is limited to a few studies.^26^ Given that current food retail interventions have failed to move the needle on significantly reducing disparities in diet and food insecurity, alternative approaches are needed.

The focus on interventions to build new supermarkets or improve convenience/corner stores (e.g., introduce fresh produce) is largely a product of food environment research that has categorized healthy food environments as those with more supermarkets and unhealthy environments as those with more corner and convenience stores.^27^ However, this dichotomy ignores the spectrum of food retail outlets that exist outside of these 2 models.^28^ For example, several recent studies have highlighted the unique contributions of small grocery stores, including coops,^29^ ethnic grocery stores,^30–32^ and discount grocery stores^33^ to food access in neighborhoods with lower income. The current literature is also limited in that most studies examining the association between store choice and food security, or diet quality, have failed to account for different store types other than supermarkets or convenience stores. Notably, most studies grouped small grocery stores with convenience or corner stores^34,35^ and sometimes included dollar or specialty stores in this category as well.^36^ In other cases, small grocery stores, sometimes also referred to as limited assortment stores^37^ or discount grocery stores, are included in the same category as supermarkets or supercenters.^38^ Thus, many food environment studies have not examined independent or small grocery stores separately when assessing associations with diet, food security, or other related outcomes. Combining store types that most consumers perceive as different may have obscured associations or led to mixed findings. The study authors are only aware of 1 previous study that looked at these associations and separated small/independent grocery stores^39^; however, this study was limited to African American women in 1 northern US city. To identify possible new food retail interventions, more exploratory research is needed to understand the role that food store choices and perceived environments play in diet and food security.

Looking at perceptions of the food environment is important because there is a mixed relationship between objectively measured food availability and diet.^40,41^ Objective measures may not capture all dimensions of food access (e.g., affordability, acceptability).^42^ Furthermore, where consumers choose to shop, which is not always the closest store, may be more predictive of dietary outcomes.^43^ While qualitatively, perceptions of the food environment are thought to be important for determining food shopping and dietary behaviors,^25^ the relationship between diet-related behaviors and objective and perceived measures of the food environment is complex,^44,45^ and both types of measures are important. For the current research, participants’ primary food shopping location was collected using the Perceived Nutrition Environment Measures Survey (NEMS-P).^46^ Thus, rather than having researchers define the type of store participants were shopping at using commercial databases or a specific metric (i.e., square footage, cash registers) as is done in some objective studies, this measure allows respondents to self-categorize their primary grocery shopping location. This approach also helps avoid bias in commonly used food store classification systems based on the racial composition of neighborhoods.^47^ Extensive pilot and psychometric testing in multiple communities showed that most consumers found answering this question easy, and their perceptions aligned with objective categorizations; pilot participants viewed supermarkets as larger, corporate stores and small grocery stores as small neighborhood stores with less variety, such as Aldi, co-op, or Trader Joe’s. They also include mom-and-pop stores in this category, but emphasized that they were generally more convenient than a grocery store.^46^

The objective of this paper is to use a novel method for categorizing food stores and quantitatively characterize the relationships between primary grocery shopping location (i.e., primary store type) and (1) demographics, (2) food security status, (3) F&V consumption, (4) perceptions of the food environment, and (5) online shopping. We will also explore the role of store perceptions on diet and food security. For this cross-sectional study, we analyzed baseline survey data from participants who were recruited as part of an intervention study focused on mobile markets, referred to as the Veggie Van study.^48^ The Veggie Van study was approved through expedited review by the University at Buffalo Institutional Review Board.

## METHODS

### Study Design and Participants

The Veggie Van study recruited 9 community-based organizations across 4 states (New York, North Carolina, Ohio, Georgia) to participate in a cluster-randomized controlled study evaluating mobile markets.^48,49^ Community-based organizations served as study partners and facilitated the recruitment of participants, along with implementing a mobile market intervention for 12 months. Details of the study recruitment and data collection methods are published elsewhere.^48,49^ Briefly, the organizations were selected because they were serving a primarily urban, a population with lower income. Partner organizations identified a total of 33 community sites, including libraries, community centers, and schools, in which participants were recruited; these communities were identified as needing more affordable access to fresh F&Vs. Study participants were recruited via each partners’ existing community engagement strategies and programming, as well as recruitment through mailing lists and social media channels. Partners recruited individuals who were interested in shopping at a mobile market using an interest form process that was identical between both the control and intervention sites; interested community members provided their contact information to the partner organization to receive updates about the proposed mobile market and were given the option to consent to being contacted by the University at Buffalo for research. The partner organizations provided the contact information of consenting individuals to the Veggie Van study team. The study team called prospective participants to screen, recruit, and verbally obtain consent over the phone.

To be eligible to participate in the Veggie Van study, individuals had to be aged ≥ 18 years, speak English or Spanish, live near or frequently visit a community site for the study (i.e., a community identified as having limited access to fresh F&Vs), and be the primary grocery shopper for their household. Recruitment methods focused on individuals with lower income and ensured that at least 60% of eligible sign-ups were from individuals participating in government assistance programs.^48^ We used baseline data from all 699 individuals participating in the Veggie Van study for the current analyses.

### Data Collection and Measures

Data collection methods are described in detail elsewhere.^48^ Briefly, all participants completed an approximately 30-minute phone survey in English or Spanish. Measures used for this analysis are described below.

#### Self-reported demographic measures.

Included age, sex, race/ethnicity, household income, marital status, education, employment status, and government assistance participation in the past 12 months (e.g., *Supplemental Nutrition Assistance Program, Special Supplemental Nutrition Program for Women, Infants, and Children, Medicaid, Temporary Assistance for Needy Families, Head Start*, etc.) with categories shown in Table 1.^48^ Education and income were collected as a categorical variable for ease of participant reporting, but converted to continuous variables for analyses to more clearly report averages by store type. Bounded midpoint estimates were created as follows for education years (≤ eigth grade = 8 years, some high school = 10 years, high school graduate 12 years, trade or beauty school graduate = 13 years, some college = 14 years, college graduate = 15 years and more than college = 18 years) and income (≤ $10,000 = $10,000, $10,000 to $19,999 = $15,000, $20,000 to $29,999 = $25,000, $30,000 to $39,000 = $35,000, $40,000 to $49,999 = $45,000, $50,000 to $59,000 = $55,000, ≥ $60,000 = $60,000). Total mouths to feed within the household (children and adults) and length of residency were also collected as components of demographic data.

#### Primary grocery shopping location (i.e., primary store type).

This variable was measured using 1 question based on the NEMS-P,^46^ but with expanded options based on previous research. Participants were asked the following: At which type of store do you buy most of your food? (with options read to participants including: a supermarket or large grocery store (examples added based on state), a supercenter such as Walmart or Target, a buying club like Sam’s Club, a small grocery store such as Aldi, a dollar store, a corner store/gas station/convenience store, a farmers’ market/farm stand/community support agriculture, a mobile produce market, or other). Participants were asked to make their own determination as to the type of store they shopped at but if they were unsure how to determine if their store was a supermarket, small grocery store or convenience store, participants were given the following descriptions based on the Illinois Prevention Research Centers Food Store *Supplemental Nutrition Assistance Program* study guidelines^50^: a supermarket sells fresh meat (e.g., beef, pork, chicken, turkey) and has ≥ 4 cash registers and at ≥ 2 of the following service counters: bakery, butcher, or deli; a small grocery store sells fresh meat (e.g., beef, pork, chicken, turkey) but does not meet all the criteria for supermarket. Examples of small grocery stores include Trader Joe’s, Aldi, Save A Lot, and some ethnic and mom-and-pop food stores; convenience stores include small, independently-owned or chain stores that sell an edited selection of staple groceries and other convenience items (i.e., ready-to-heat and ready-to-eat foods). They often sell fresh milk and may have a deli or sell some processed meats (hot dogs, cold cuts, etc.) and other hot foods. Convenience stores are typically open long hours, and some sell gasoline as well. Respondents were asked to provide the names of the 2 places where they purchased most of their food. Of those two, they were asked which place they purchased more food from; this was used as their primary store.

#### Shopping behavior and perceptions.

The primary store type used questions from the NEMS-P (e.g., shopping frequency, transportation, distance, reason for shopping).^46^ Price, quality, and variety at stores were measured using a 4-point Likert scale ranging from a score of 1 (i.e., very affordable, very high quality, very high variety) to 4 (very expensive, very low quality, very low variety). After the onset of the coronavirus 2019 pandemic in 2020, we added the question: In the past year, have you purchased groceries online? This refers to purchasing food online from a local store and picking it up at the store or having it delivered.

#### Perceived access to F&V.

This variable was measured using the NEMS-P and included 3 3-item scales, which are designed to be summed for a score of 3–15; this scale was validated to assess price, quality, and availability of F&V within 1-mile or a 30-minute walk of an individual’s home.^51^ We repeated this scale to also measure their perceptions of food access in general.

#### Food security.

This variable was measured using the US Department of Agriculture’s US Adult Food Security Survey Module.^52^ Survey responses are coded to produce a food security score on a scale of 0–10. For describing the sample, food security status was categorized as: high food security (0), marginal food security (1–2), low food security (3–5), and very low food security (6–10).^52^

#### Fruit and vegetable intake.

This variable was calculated as the mean times per day over the past month (i.e., times per day) from the 2013 Behavioral Risk Factor Surveillance Survey, which includes 6-items measuring frequency of consuming fruit or fruit juice (2-items) and vegetables including leafy greens, orange-colored vegetables, beans, and other vegetables (4-items); fried potatoes were excluded.^53^

### Analysis

All analyses were completed using SAS (version 9.4, SAS Institute Inc, 2013). We conducted 2-way chisquare tests of indenpendence to examine differences in frequencies of categorical demographic variables (gender, race, ethnicity, government assistance) by primary store type. Fisher’s exact test was used to examine differences in food store perceptions by primary store type because of small numbers in some cells. Analysis of variance was used to examine differences in continuous demographic variables (education, age, household size, income), perceived access, food security score, and F&V intake by primary shopping location. In addition, we used analysis of covariance (ANCOVA) to look at the relationship between food security, F&V intake, and online shopping. Analysis of covariances looking at primary store type, controlled for education, as it was the only demographic variable shown to be associated with this variable. Sensitivity analyses were conducted using additional demographic variables associated with diet and food security, but this did not change the results. Omnibus significant differences among primary store types using ANCOVA were followed by *post-hoc* orthogonal contrasts by store type. While they are included in descriptive analyses of the full sample, participants who reported their primary store type as corner/convenience stores (n = 2), farmers’ markets/community support agriculture (n = 2), mobile markets (n = 1), or other (n = 3) were excluded from further analyses because of small sample size. While dollar stores were included as a possible primary store type, no one selected this option.

## RESULTS

### Participant Characteristics

Participants (n = 699) had a mean age of 48.2 ± 15.0 years and a mean household size of 2.8 ± 1.6 people. The majority of the sample (84.3%) reported being female (Table 1). The most reported race was Black or African American (46.4%), and most people reported an income of less than $40,000 per year (60.3%) and at least some college education (67.6%). Approximately 65.2% of participants reported receiving some form of government assistance.

### Shopping Behaviors

Supermarkets were most commonly reported as participants’ primary store type (67.5%), followed by supercenters (16.2%), small grocery stores (13.2%), supercenters (16.2%) and buying clubs (2.0%). Most (61.8%) respondents reported that they did not shop for groceries online; 16.2% of participants said they shopped online a few times in the past year, 11.4% said they shopped online about once per month, and 10.6% were shopped online every week or almost every week. Participants rated the quality of food available at a food store as having the highest importance when deciding which stores to shop at (rated 3.76 out of 4.00), followed by price (3.75), selection (3.68), and proximity to home (3.54).

### Differences in Demographics by Primary Store Type

There were no statistically significant differences by age, gender, or race in participants’ primary grocery shopping location (data not shown). There were differences in primary store type by education level (*P* = 0.04); People who primarily shopped at supermarkets on average had higher levels of education than those who shopped at supercenters (*P* = 0.03) and less education than those who shopped at buying clubs (*P* = 0.03). People who shopped at supercenters had less education than those who shopped at buying clubs (*P* = 0.003) and small grocery stores (*P* = 0.02). People who shopped at supermarkets also reported significantly higher (*P* = 0.02) average household income ($33,374) than those who shopped at supercenters ($28,398).

### Differences in Perceptions of the Food Environment by Primary Store Type

There were no statistically significant differences in perceived access to fresh F&V based on primary store type (data not shown). There were, however, differences in perceptions of the affordability of F&Vs at their primary grocery shopping location based on store type (*P* = 0.003) (Table 2). *Post-hoc* orthogonal contrasts controlling for education indicated that people who primarily shopped at small stores viewed their primary shopping location as more affordable than supermarket shoppers (*P* < 0.001). There were no differences in ratings of quality or variety based on primary store type (Table 2).

### Differences in Food Security, Diet, and Online Shopping by Primary Store Type

Overall, 34.5% of participants reported low or very low food security (Table 1), and average past-month F&V intake was 3.23 ± 2.27 times/d (n = 684) (data not shown). Associations between primary store types and food insecurity, past-month fruit intake, and past-month vegetable intake are shown in Table 3. After controlling for education, average past-month vegetable intake significantly differed (*P* = 0.04) by primary store type. *Post-hoc* analyses indicated a significant difference between participants who primarily shopped at supercenters (e.g., Walmart or Target) vs supermarkets (*P* = 0.04) or small grocery stores (*P* = 0.006). There were no statistically significant differences in online shopping by primary store type (data not shown). There were no statistically significant associations between perceptions of quality and variety at participants’ primary store type and past-month F&V intake (Table 4). However, greater perceived F&V affordability was associated with higher past-month vegetable intake (*P* = 0.01).

## DISCUSSION

This study fills a gap in the literature by exploring how where people choose to shop is related to their diet, food security score, and perceptions of the food environment. Notably, we found that the participants’ primary grocery shopping location was associated with vegetable consumption, but not fruit consumption or food security. People who report that a small grocery stores as their primary store type had the highest vegetable intake (2.00 times/d), which is above the median national vegetable intake of 1.70 times/d.^7^ Those who shopped at supercenters reported the lowest intake (1.52 times/d), which is below the national median. In contrast, an earlier study conducted in Detroit and surrounding suburbs examined associations between where people shop and diet across multiple store types and found that women shopping at chain supermarkets consumed 1.22 times more F&Vs than those shopping at independent grocery stores. They also found that independent grocery stores were rated higher in selection, quality, and affordability than chain supermarkets.^54^ While our study did not specifically look at independent stores separate from chains, many of the small grocery stores reported in the study were independent stores, and most small grocery store shoppers said they shopped at Aldi. While the previous study compared independent stores with supermarkets, our study compared additional store types, including supercenters, smaller grocery stores, and buying clubs. In addition, this study improved generalizability compared with previous research by including participants from 33 communities across 4 US states.

The findings from this study and previous literature point to reasons why shoppers at small grocery stores may be eating more vegetables. First, we observed that participants in our study, whose primary grocery shopping location was a small grocery store, rated their primary store as having more affordable F&V than shoppers at supermarkets. Furthermore, participants who rated their primary store as having more affordable fresh F&Vs had significantly higher F&V intake. Our findings on the affordability of produce in small stores are in line with several studies showing that small grocery stores have lower prices,^55,56^ but contrary to findings from a case study in Hartford, Connecticut that used objective scoring and found that F&V prices were lower in larger stores.^33^ While we did not find a relationship between where people shopped and how they rated F&V quality, we did find that F&V quality was a top reason why participants chose to shop at their current store. Although not statistically significant, we saw a consistent pattern indicating that higher perceptions of fresh F&V quality at the primary store may be associated with higher F&V intake. This finding is in line with previous research emphasizing that produce quality is a top priority for consumers with lower income when choosing where to buy F&V, perhaps even more so than price.^22,25^ Research in Scotland objectively rated produce quality in small, medium, and large stores and found that medium stores, on average, had the highest quality F&Vs.^57^ A recent multistate case study examined small stores that served primarily communities with lower income across the US.^31^ Notably, these studies found that a key to success among small grocery stores was that they were highly engaged with their customers and responsive to community preferences.^58^

Overall, research looking at the quality and price of food generally and fresh F&Vs specifically at small stores has been mixed. A general limitation of this body of research has been conflicting definitions of small grocery stores. This study is unique in that data came from self-reported food shopping behaviors and perceptions of the food environment, rather than objective measures. To do this, we used validated measures of the participants’ perceptions of the food environment that include discrete store types that match how consumers think about where they shop, including supercenters, supermarkets, small grocery stores, and buying clubs.^46^ Notably, this study is one of the only studies examining diet and food security to designate small grocery stores as a unique entity separate from supermarkets and corner stores. While this research separated out small grocery stores, it did not differentiate between independent and chain small grocery stores.

This study also has several limitations. This research took place among residents of primarily urban underserved communities, which are often targets for food access initiatives and likely cannot be generalized to suburban or rural neighborhoods, which have notably different food environments.^55,59,60^ Furthermore, participants in this study had to be interested in more food options in their community, specifically shopping at a mobile market. While this may bias the sample toward people who had an interest in a mobile market, very few people were excluded because of this eligibility criterion. Furthermore, as this is a cross-sectional study, it is difficult to know if people with certain behaviors (e.g., high F&V consumption) chose to shop at certain stores or if it is the store that impacts their behavior. Interestingly, a previous study tried to address this question by looking at whether the retail food environment or individual characteristics had more impact on F&V intake and found comparable contributions from both factors.^61^ To address potential demographic confounders, such as education level, that differed by primary grocery shopping location, we controlled for this after finding a significant association with vegetable intake. While looking at education as a continuous variable allowed us to improve the interpretation of results and reduce degrees of freedom in the analyses, it may have incorrectly categorized individuals’ years for education, particularly for those with postgraduate degrees, as these can vary in length. However, this was a relatively small portion of the population (15%).

A challenge to the interpretation of these findings is the small number of participants who reported mainly shopping at small grocery stores and buying clubs; It is unclear if this is due to less availability of these stores or a preference for supermarkets. Another limitation of this research is that we did not directly measure the food environment but rather relied on individuals’ perceptions of their primary store type. Perceptions, however, may have a greater impact on behavior than objectively measured food environment factors.^62^ Researcher classification can be problematic because common food store classifications, such as Dun & Bradstreet and InfoUSA, are informed by store attributes such as the number of cash registers and the presence of certain food categories (e.g., produce) or sections (e.g., butcher); however, food stores, particularly atypical stores and stores in predominantly Black communities, may be misclassified.^47^ These classifications also do not fully capture dimensions of access or healthfulness. Finally, data collection for this study occurred between 2020 and 2022, with many sites collecting data during the coronavirus disease 2019 pandemic, when there were significant fluctuations in food security, and people may have changed their shopping behaviors.^63^ These factors may have limited the ability to see differences in food security by primary store type.

## IMPLICATIONS FOR RESEARCH, PRACTICE, AND POLICY

On the basis of this research, we recommend more qualitative research to help explain whether shopping choices are driven by preferences or access. Longitudinal mediation analyses or intervention studies manipulating produce prices and quality could also help to determine the exact role that F&V affordability plays in determining dietary intake. In addition, it may be helpful to distinguish independent small grocery stores from discount chain stores in addition to supermarkets and corner stores. Black et al^64^ created a tool to distinguish these stores, which could be employed for this type of research. Our research suggests that initiatives designed to improve diet in communities with lower income should consider small grocery stores in addition to supermarkets and corner stores. While there is 1 natural experiment in progress,^29^ we are not aware of any published studies that have examined the impact of building new small grocery stores in underserved communities.

## Figures and Tables

**Table 1.** Demographic Characteristics of the Veggie Van study Participants (n = 699)

Characteristics	n (%)
Gender	
Male	107 (15.3)
Female	589 (84.3)
Other/refused/missing	3 (0.4)
Marital status	
Married or living with a partner	241 (34.5)
Single	317 (45.4)
Divorced	76 (10.9)
Separated/widowed	61 (8.7)
Refused/missing	4 (0.5)
Education	
Less than eighth grade	13 (1.9)
Some high school/high school grad/GED	172 (24.6)
Trade or beauty school graduate	38 (5.4)
Some college	170 (24.3)
College graduate	198 (28.3)
Postgraduate	105 (15.0)
Refused/missing	3 (0.4)
Annual household income, $	
< 10,000	122 (17.5)
10,000–19,999	120 (17.2)
20,000–29,999	89 (12.7)
30,000–39,999	90 (12.9)
40,000–49,999	57 (8.2)
50,000–59,999	53 (7.6)
≥ 60,000 or more	124 (17.7)
Missing/refused	44 (6.3)
Hispanic or Latino/Latina	69 (9.9)
Race	
American Indian or Native American	6 (0.9)
Asian	14 (2.0)
Black or African American	324 (46.4)
Multiracial	27 (3.9)
Native Hawaiian or Pacific Islander	2 (0.3)
White	281 (40.2)
Refused/missing	45 (6.4)
Age, years	
18–24	22 (3.3)
25–34	133 (20.0)
35–44	132 (19.8)
45–64	269 (40.5)
65–84	105 (15.8)
85–99	3 (0.5)
> 100	1 (0.2)
Refused/missing	34 (4.9)
Government assistance	
Receiving any government assistance	456 (65.2)
WIC	70 (10.0)
SNAP	269 (38.5)
Free or Reduced-price school breakfast or lunch	175 (25.0)
Head Start	34 (4.9)
Medicaid	283 (40.5)
TANF or Welfare	29 (4.1)
Social Security disability benefits	176 (25.2)
None	207 (29.6)
Refused/missing	36 (5.2)
Food Security Status	
High food security (score = 0)	315 (45.1)
Marginal food security (score = 1–2)	140 (20.0)
Low food security (score = 3–5)	126 (18.0)
Very low food security (score = 6–10)	115 (16.5)
Refused/missing	3 (0.4)

GED, indicates General Educational Development; SNAP, Supplemental Nutrition Assistance Program; TANF, Temporary Assistance for Needy Families; WIC, Special Supplemental Nutrition Program for Women, Infants, and Children.

**Table 2.** Differences in Shoppers’ F&V Perceptions at their Primary Grocery Shopping Location

Variables	Supermarket	Supercenter	Buying Club	Small Grocery Store
Price of fresh F&Vs^a^				
Very affordable	15.7 (73)	19.6 (22)	7.1 (1)	34.8 (32)
Somewhat affordable	47.0 (218)	55.4 (62)	50.0 (7)	42.4 (39)
Somewhat expensive	30.8 (143)	20.5 (23)	42.9 (6)	14.1 (13)
Very expensive	6.5 (30)	4.5 (5)	0	8.7 (8)
Quality of fresh F&Vs^b^				
Very high quality	23.2 (107)	19.6 (22)	14.3 (2)	23.1 (21)
Somewhat high quality	53.7 (248)	47.3 (53)	57.1 (8)	52.8 (48)
Somewhat low quality	21.0 (97)	28.6 (32)	21.4 (3)	20.9 (19)
Very low quality	2.2 (10)	4.5 (5)	7.1 (1)	3.3 (3)
Variety of fresh F&Vs^c^				
Very high variety	27.6 (129)	21.4 (24)	28.6 (4)	26.4 (24)
Somewhat high variety	47.5 (222)	48.2 (54)	50.0 (7)	37.4 (34)
Somewhat low variety	18.8 (88)	24.1 (27)	14.3 (2)	33.0 (30)
Very low variety	6.0 (28)	6.3 (7)	7.1 (1)	3.3 (3)

F&Vs indicate fruits and vegetables.

a Fisher’s Exact Test, *P* = 0.003;

b Fisher’s Exact Test, *P* = 0.56;

c Fisher’s Exact Test, *P* = 0.21.

Note: All values are presented as n (%).

**Table 3.** Differences in Food Security and F&V Consumption by Primary Grocery Shopping Location

Primary Grocery Shopping Location	n	Mean ± SD	F Value, *P*
Food security score (0–10)^a^			0.96, 0.41^a^
Supermarket or large grocery store	469	2.10 ± 2.76	
Supercenter	112	2.42 ± 2.76	
Buying Club	14	1.29 ± 1.86	
Small grocery store	92	2.30 ± 2.80	
Past-month vegetable intake (times/d)^b^			2.78, 0.04^b^
Supermarket or large grocery store	471	1.82 ± 1.21	
Supercenter	112	1.52 ± 1.13	
Buying Club	14	1.82 ± 1.60	
Small grocery store	92	2.00 ± 1.28	
Past-month fruit intake (times/d)^a^			0.17, 0.92^a^
Supermarket or large grocery store	469	1.10 ± 0.99	
Supercenter	111	1.05 ± 1.20	
Buying Club	14	1.12 ± 1.53	
Small grocery store	92	1.04 ± 0.85	

F&Vs indicate fruits and vegetables.

a Analysis of variance;

b Analysis of covariance was used to control for education as the main effects were found to be statistically significant. *Post-hoc* orthogonal contrasts found statistically significant differences in vegetable intake between supermarkets and supercenters (*P* = 0.02) and small grocery stores and supercenters (*P* = 0.006).

Note: Higher Food Security Scores indicate poorer food security. A score of 0 indicates food secure.

**Table 4.** Differences in F&V Intake Based on Perceptions of Price, Quality, and Variety at Their Primary Grocery Shopping Location

Variables	Average Past-Month F&V Intake	Average Past-Month Vegetable Intake	Average Past-Month Fruit Intake
Price of fresh F&Vs			
Very affordable (n = 128)	3.62 ± 1.79	2.13 ± 1.27	1.50 ± 1.10
Somewhat affordable (n = 320)	3.21 ± 2.27	1.76 ± 1.25	1.44 ± 1.45
Somewhat expensive (n = 182)	3.19 ± 2.66	1.73 ± 1.11	1.46 ± 1.97
Very expensive (n = 46)	2.69 ± 1.70	1.58 ± 1.04	1.10 ± 0.89
F value, *P*	2.12, 0.10	3.93, 0.009	0.82, 0.48
Quality of fresh F&Vs			
Very high quality (n = 154)	3.56 ± 2.88	1.95 ± 1.26	1.90 ± 2.09
Somewhat high quality (n = 359)	3.28 ± 2.17	1.82 ± 1.27	1.44 ± 1.39
Somewhat low quality (n = 152)	2.94 ± 1.82	1.61 ± 1.03	1.32 ± 1.22
Very low quality (n = 21)	2.50 ± 1.51	1.68 ± 1.18	0.79 ± 0.47
F value, *P*	2.6, 0.05	2.16, 0.09	2.08, 0.10
Variety of fresh F&Vs			
Very high variety (n = 182)	3.28 ± 2.04	1.88 ± 1.18	1.40 ± 1.24
Somewhat high variety (n = 312)	3.16 ± 1.99	1.76 ± 1.20	1.39 ± 1.20
Somewhat low variety (n = 147)	3.43 ± 3.08	1.80 ± 1.30	1.63 ± 2.36
Very low variety (n = 37)	2.96 ± 1.83	1.75 ± 1.16	1.15 ± 1.03
F value, *P*	0.70, 0.56	0.42, 0.74	1.30, 0.27

F&Vs indicate fruits and vegetables.

Note: All values are presented as mean time/d ± SD. All analyses use analysis of variance.
